# Salivary Microbiome Diversity in Caries-Free and Caries-Affected Children

**DOI:** 10.3390/ijms17121978

**Published:** 2016-11-25

**Authors:** Shan Jiang, Xiaoli Gao, Lijian Jin, Edward C. M. Lo

**Affiliations:** Faculty of Dentistry, The University of Hong Kong, Prince Philip Dental Hospital, 34 Hospital Road, Sai Ying Pun, Hong Kong, China; shanj@hku.hk (S.J.); ljjin@hku.hk (L.J.); hrdplcm@hku.hk (E.C.M.L.)

**Keywords:** etiology, 16S rRNA gene, early childhood caries, microbiome, saliva, children

## Abstract

Dental caries (tooth decay) is an infectious disease. Its etiology is not fully understood from the microbiological perspective. This study characterizes the diversity of microbial flora in the saliva of children with and without dental caries. Children (3–4 years old) with caries (*n* = 20) and without caries (*n* = 20) were recruited. Unstimulated saliva (2 mL) was collected from each child and the total microbial genomic DNA was extracted. DNA amplicons of the V3-V4 hypervariable region of the bacterial 16S rRNA gene were generated and subjected to Illumina Miseq sequencing. A total of 17 phyla, 26 classes, 40 orders, 80 families, 151 genera, and 310 bacterial species were represented in the saliva samples. There was no significant difference in the microbiome diversity between caries-affected and caries-free children (*p* > 0.05). The relative abundance of several species (*Rothia dentocariosa*, *Actinomyces graevenitzii*, *Veillonella* sp. *oral taxon 780*, *Prevotella salivae*, and *Streptococcus mutans*) was higher in the caries-affected group than in the caries-free group (*p* < 0.05). *Fusobacterium periodonticum* and *Leptotrichia* sp. *oral clone FP036* were more abundant in caries-free children than in caries-affected children (*p* < 0.05). The salivary microbiome profiles of caries-free and caries-affected children were similar. Salivary counts of certain bacteria such as *R. dentocariosa* and *F. periodonticum* may be useful for screening/assessing children’s risk of developing caries.

## 1. Introduction

Dental caries (tooth decay) is a highly prevalent chronic childhood disease worldwide [1]. It is a significant source of pain and impairs the health-related quality of life for many children and may result in serious infection, hospitalization, and even fatality under some circumstances [2]. It is well noted that the bacteria in dental biofilms play an essential role in the initiation and progression of caries [3]. *Streptococcus mutans* is considered the most cariogenic of all oral streptococci [4]. Nevertheless, it can be detected in plaque samples from some caries-free children, whereas some subjects with severe early childhood caries do not have any detectable *S. mutans* [5]. These findings suggest that *S. mutans* is not the only cariogenic bacteria and other bacterial species might be responsible for caries initiation and development. For instance, the presence and levels of Candida albicans in saliva was reported to be strongly associated with caries pathogenesis, particularly in children, adolescents, and young adults [6,7,8]. In addition, *Campylobacter showae*, *Parvimonas micra*, and *Leptotrichia hofstadii* may be considered as potential biomarkers for dental caries [9].

It is estimated that more than 700 bacterial species or phylotypes exist in the oral cavity, of which over 50% cannot be successfully cultivated, even with advanced bacterial cultivation techniques [10]. It is implied that the etiology of dental caries remains unclear, partially because of our limited understanding of the oral microbial world [11,12]. Recently, culture-independent methods based on 16S rRNA gene analysis have made it possible to detect those uncultivable species and investigate the diversity of oral microorganisms [13,14]. A few studies have investigated the association between dental caries and the diversity of oral microbiome in different populations using 16S rRNA gene sequencing methods. A study evaluated the oral microbial diversity of children aged 6–8 years with mixed dentition and found that the microbe diversity within saliva derived from children with active dental caries was higher than that in caries-free children [9]. However, Yang et al. [11] examined the oral microbiota diversity in saliva among 18–22 year old undergraduates with permanent dentition and suggested that there was a similar diversity among subjects with or without active caries. In another study, a longitudinal analysis of saliva samples collected from 72 infants showed reduced bacterial diversity as caries progressed from sound teeth to cavitated lesions [15].

The existing findings on the association of bacterial diversity with dental caries remains highly controversial. This study aimed to phylogenetically characterize and compare the salivary microbiome diversity of preschool children with or without dental caries, and identify potential species associated with caries by means of 16S rRNA gene pyrosequencing based on Illumina MiSeq platform.

## 2. Results

### 2.1. Features of Salivary Microbiomes

16S rRNA gene amplicons from saliva samples of caries-affected (*n* = 20) and caries-free (*n* = 20) children were sequenced with Illumina MiSeq technology. A total of 16,810,339 raw sequences with an average length of 467 bp were produced. After trimming, 770,777 gene reads of 16S rRNA with an average length of 466 bp were obtained. The species-level operational taxonomic units (OTUs) at 3% dissimilarity for each sample are shown in Table 1. A total of 91,501 OTUs were obtained from these 40 samples, with 1559 to 2770 OTUs per sample, using the Uclust program [16]. Altogether, 17 phyla, 26 classes, 40 orders, 80 families, 151 genera, and 310 bacterial species were represented by all the samples. There was no significant difference in salivary microbial flora profiles between boys and girls in each group (*p* > 0.05).

### 2.2. Microbiome Diversity and Richness

**Figure 1:**
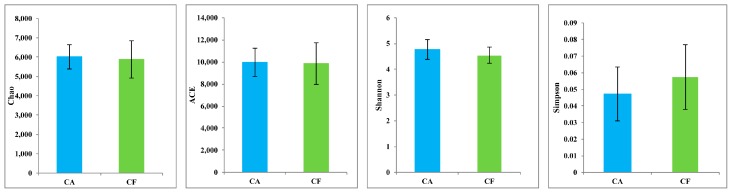
Phylogenetic diversity (mean ± SD) of the salivary microbiomes of the caries-affected (CA) and caries-free (CF) children.

**Figure 2:**
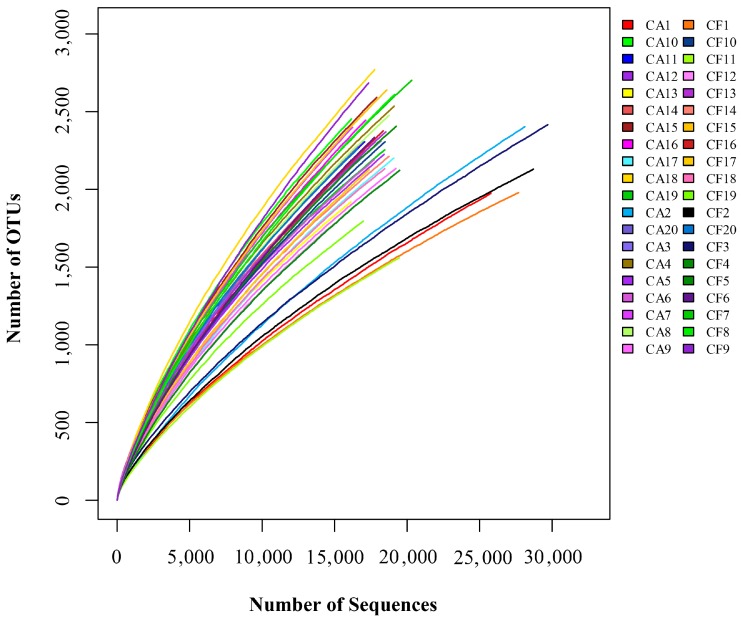
Rarefaction curves for the caries-affected (CA) and caries-free (CF) children.

The species-level OTUs and species diversity and richness estimates were calculated for each sample. There was no significant difference between the number of OTUs at 3% dissimilarity of the caries-affected (mean OTUs = 2316) and caries-free (mean OTUs = 2259) children. The richness of salivary microbiota was measured by the Chao and Abundance-based Coverage Estimator (ACE), while the diversity of the salivary microbiome was measured by the Shannon and Simpson diversity indices. There were no significant differences in the richness and diversity indices between the caries-affected and the caries-free children (*p* > 0.05) (Figure 1). In addition, comparisons of the rarefaction curves revealed that the two groups had an overall similar richness of OTUs at the 3% dissimilarity level (Figure 2).

### 2.3. Community Structures

**Figure 3:**
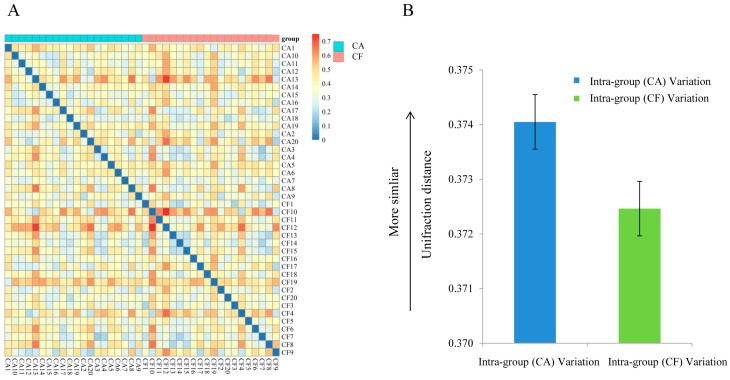
Comparison of community structures within and between the caries-affected (CA) and caries-free (CF) groups. (**A**) Heatmap of the weighted UniFrac distance within and across the CA and CF groups; (**B**) The weighted UniFrace distance values (mean ± SD) of the CA and CF groups.

The community structures within the microbiotas were compared using FastUniFrac [17] for paired comparisons on the distances between two microbiotas in regard to the fraction of evolutionary history that separates the organisms (Figure 3A). As shown in Figure 3B, there was no significant difference of the degree of variations between the caries-affected and caries-free children (*p* > 0.05).

### 2.4. Core Salivary Microbiome

**Figure 4:**
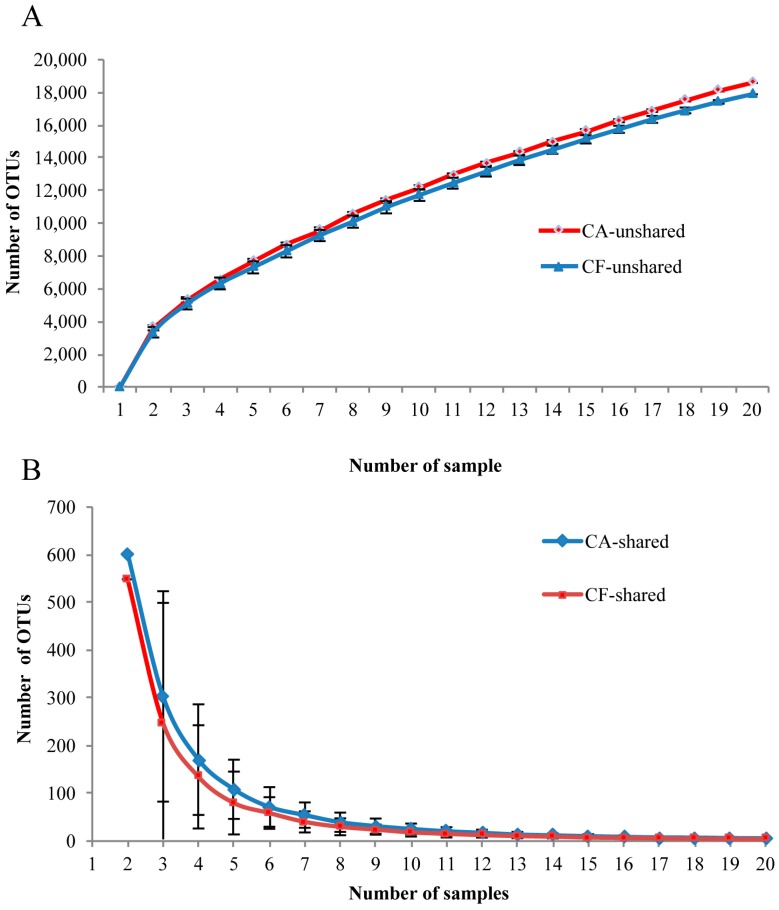
Curves of shared and unshared OTUs (mean ± SD) among children in the caries-affected (CA) and caries-free (CF) groups. (**A**) Unshared OTUs among children in the CA and CF groups; (**B**) Shared OTUs among children in the CA and CF groups.

**Figure 5:**
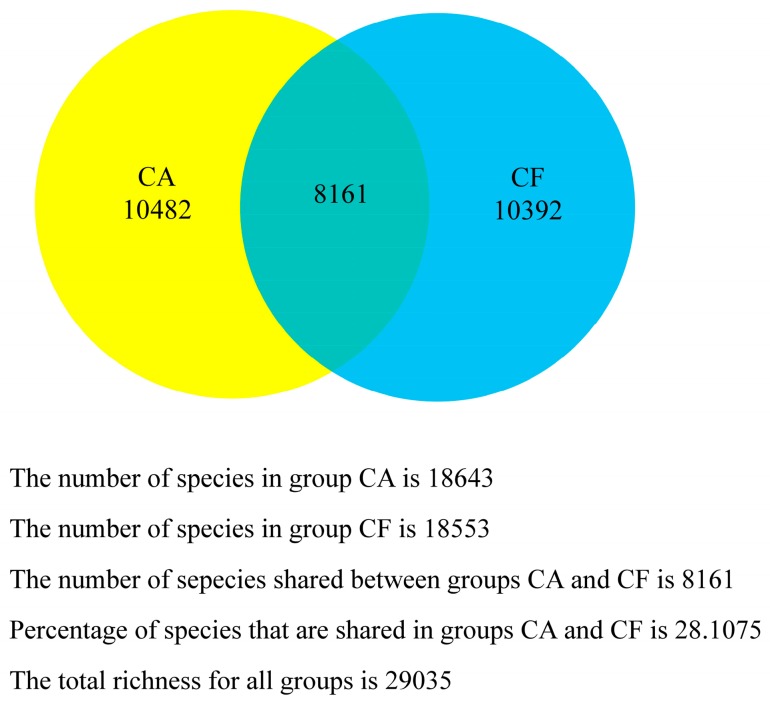
Venn diagram of the number of OTUs shared and unshared in the caries-affected (CA) and caries-free (CF) groups.

To investigate whether or not there is a core salivary microbiome across all subjects, the shared and unshared OTUs were analyzed. The shared OTU curves revealed a gradual decrease of OTU sharing with each additional sample in both groups (Figure 4A). Namely, the shared OTUs were gradually reduced with samples from either the caries-free group or the caries-affected group. Both of the unshared OTU curves revealed a slow increase of OTU sharing with the individual additional sample (Figure 4B). In addition, as shown in Figure 5, the caries-free and caries-affected groups presented 29,035 OTUs with 8161 shared OTUs, while 18,553 and 18,643 OTUs were unique to the respective groups. As for comparisons within the groups, four OTUs (0.14%) including OTU1, OTU5, OTU6, and OTU369, and six OTUs (0.21%) including OTU1, OTU2, OTU196, OTU264, OTU273, and OTU362 were present in all 20 samples of the caries-free and caries-affected groups, respectively. Furthermore, no OTU was detected in all 20 samples of the caries-free group but not in any sample of the caries-affected group, and vice versa.

### 2.5. Comparison of Bacterial Composition between the Caries-Affected and Caries-Free Groups

The microbial compositions of the caries-affected and caries-free children were compared. At the phylum level, 15 phyla were identified in the caries-affected group, while 11 phyla were identified in the caries-free group. The most abundant were *Firmicutes* (approximately 37%–61% of the total sequences in the caries-affected group and 38%–64% of the total sequences in the caries-free group), *Bacteroidetes* (approximately 7%–35% of the total sequences in the caries-affected group and 5%–41% of the total sequences in the caries-free group), *Proteobacteria* (approximately 3%–47% of the total sequences in the caries-affected group and 4%–53% of the total sequences in the caries-free group), *Actinobacteria* (approximately 6%–23% of total sequences in the caries-affected group and 4%–24% of total sequences in the healthy group), and *Fusobacteria* (approximately 0.5%–14% of the total sequences in the caries-affected group and 0.2%–12% of the total sequences in the caries-free group). These five most dominant phyla constituted 99.1% of the total sequences. However, there was no significant difference in these five phyla between the caries-affected group and the caries-free group (all *p* > 0.05).

**Figure 6:**
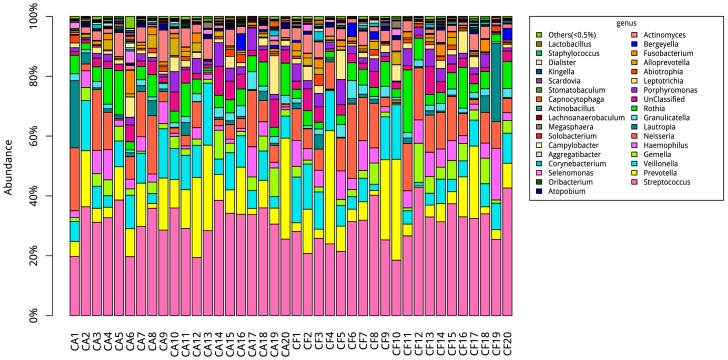
Analyses of the predominant bacterial genera in the caries-affected (CA) and caries-free (CF) groups.

At the genus level, 117 genera were found in the caries-affected group while 123 genera were found in the caries-free group. Among these genera, *Streptococcus*, *Prevotella*, *Veillonella*, *Neisseria*, *Rothia*, *Haemophilus*, and *Gemella* constituted 75% of the salivary microbial communities of the caries-affected group and 74% of the salivary microbial communities of the caries-free group (Figure 6). There was no significant difference in these eight most frequently detected genera between the two groups (all *p* > 0.05). On the contrary, the genera of *Lactobacillus* and *Scardovia* were significantly more frequently detected in the caries-affected group than the caries-free group with low relative abundance in the total sequences (both *p* < 0.05).

**Figure 7:**
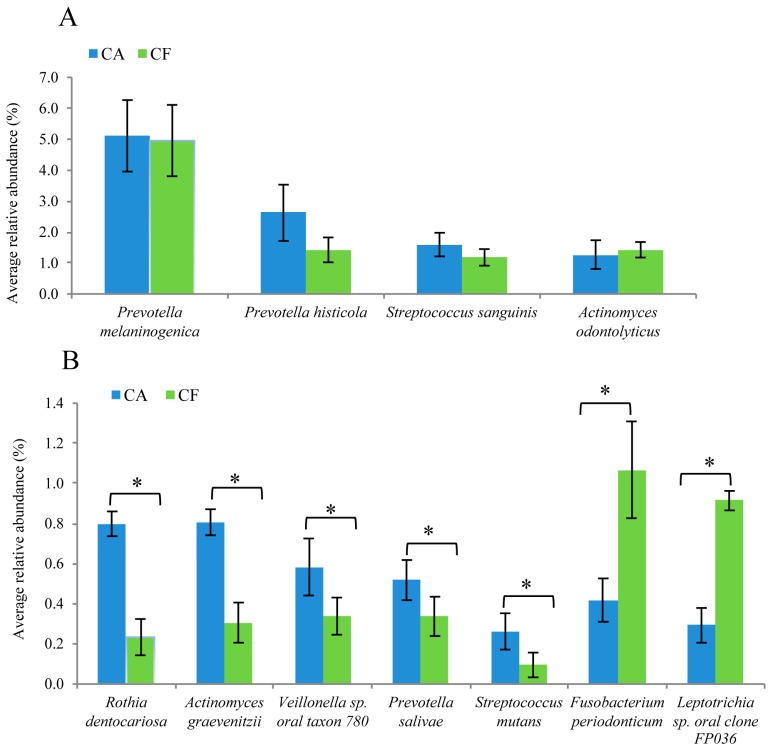
Comparison of the bacterial composition between the caries-affected (CA) and caries-free (CF) groups at the species level. (**A**) The predominant species in both CA and CF groups; (**B**) Bacterial species significantly more prevalent in the CA or CF groups (* *p* < 0.05).

At the species level, 268 and 244 species were identified in the caries-affected and the caries-free group, respectively. *Prevotella melaninogenica*, *Prevotella histicola*, *Streptococcus sanguinis*, *Actinomyces odontolyticus* were the most frequently shared species in both groups. Among these four predominant species, *P. melaninogenica*, *P. histicola*, and *S. sanguinis* were observed more frequently in the caries-affected group than the caries-free group, but the difference failed to reach statistical significance (all *p* > 0.05) (Figure 7A). Furthermore, *Rothia dentocariosa*, *Actinomyces graevenitzii*, *Veillonella* sp. *oral taxon 780*, *Prevotella salivae*, and *S. mutans* were more prevalent in the caries-affected group than in the caries-free group (all *p* < 0.05) (Figure 7B). *Fusobacterium periodonticum* and *Leptotrichia* sp. *oral clone FP036* were detected more frequently in the caries-free group than in the caries-affected group (both *p* < 0.05) (Figure 7B).

## 3. Discussion

The advent of molecular analysis methods makes it possible to investigate bacterial microbiome diversity by using DNA fingerprint techniques such as polymerase chain reaction denaturing gradient gel electrophoresis (PCR-DGGE), the Human Oral Microbe Identification Microarray (HOMIM), and bacterial 16S rRNA gene sequence analysis [12,18,19]. Among these methods, PCR-DGGE and HOMIM focus on the predominant or major species of the microbial community, while the 16S rRNA gene pyrosequencing allows the detection of comprehensive profiles of microbiome, including rarer species and not-yet-cultivated bacteria species. The investigation of a more complete composition of the oral bacterial communities is crucial for understanding the microbial etiology and pathogenesis of dental caries more deeply. In the present study, Illumina MiSeq pyrosequencing was used, which provides a powerful sequencing platform for the high-throughput, rapid, and in-depth characterization of the microbial community, to explore the composition of the oral microbiome by targeting the 16S rRNA gene hypervariable regions of V3-V4. This region provides abundant information for the taxonomic classification of communities of microbes from human microbiome samples and was applied in previous similar studies [20,21,22,23].

In the present study, a remarkably high phylogenetic diversity in both groups was observed. Moreover, the caries-affected and the caries-free children displayed an overall similar level of phylogenetic diversity at 97% identity, which is consistent with previous findings [11,12]. This is in agreement with the high degree of similarity of the microbial community structure of the microbiomes of the caries-affected and caries-free children, as suggested by the UniFrac distance metrics analysis. This observation suggests that the oral microbial diversity in saliva may not play an essential role in early childhood caries. It should be noted that the bacterial richness of the saliva samples in the present study were not yet satiated, as suggested by rarefaction curves. This indicates that the true microbial diversity of saliva in children might be greater that what was identified, as similarly found in other studies [11,24].

In addition, the existence of core salivary microbiome was tested for evaluating the conservation level of microbiota among these study subjects. In both groups, the OTU sharing gradually decreases with each individual additional sample. Furthermore, no OTU was identified in all the samples in one group but not in any samples of the other group. These indicate that the degree of shared organism lineages among individual salivary microbiome in children was minimal. This finding supports the fact that there is no species-level organismal ‘core’ of salivary microbiome correlated with dental caries in children, as suggested by a previous study [24]. However, Zaura et al. [25] found that salivary microbiome from three subjects shared 387 out of 818 OTUs and suggested that there was a “core microbiome” in the oral microbial communities. These contradictory results require further investigation.

The findings of this study show that the genera composition type, and in particular, the predominant microbiota including *Streptococcus*, *Prevotella*, *Veillonella*, *Neisseria*, *Rothia*, and *Haemophilus* in saliva from the caries-affected and caries-free children were similar, although slight differences existed between the groups. This finding generally agreed with that of a previous study [19,20], in which similar distribution at the genus level was found in dental plaque collected from severe caries and caries-free children (32 months old). The roles of both Streptococcus and Lactobacillus in dental caries are well documented [26,27,28]. It is worth mentioning that in accord with previous studies [12,19], a similar distribution of *Streptococcus* at the genus level was found in the caries-free and caries-affected groups. One possible explanation is that the genus Streptococcus contains several bacterial species, including but not limited to *S. mutans*, *S. sanguinis*, *Streptococcus oralis*, *Streptococcus sobrinus*, *Streptococcus mitis*, and *Streptococcus gordonii*, and these different species may play various roles in the process of dental caries. Although *Lactobacillus* was significantly more abundant in the caries-affected groups than in the caries-free group (0.06% vs. 0.002%, respectively), it was almost absent in all the samples. As *Lactobacilli* is more associated with the progression of caries, it is more likely to be abundant in carious lesions but not in saliva and plaque. Furthermore, we found that the genus *Scardovia*, which was newly recognized as cariogenic bacteria involved in the later stage of severe early childhood caries [29], was significantly more frequently detected in the caries-affected group than in the caries-free group, with a low relative abundance in the total sequences. This finding is consistent with that of a previous study [30].

With the advantages of the 16S rRNA gene sequencing, several uncommonly detected or not-yet-cultivated bacteria including *R. dentocariosa*, *A. graevenitzii*, *V.* sp. *oral taxon 780*, and *P. salivae* were found to be more abundant in the caries-affected group than the caries-free group. This suggests that *R. dentocariosa*, *A. graevenitzii*, *V.* sp. *oral taxon 780*, and *P. salivae* might be associated with dental caries and may possibly play a role in the development of caries. In accord with the previous studies, *R. dentocariosa* and *V.* sp. *oral taxon 780* have also been detected in people with dental caries [31,32]. *P. salivae* was isolated from the saliva of patients with chronic periodontitis [33] and *A. graevenitzii* was isolated from human respiratory tract secretions [34]. The role of *P. salivae* and *A. graevenitzii* in dental caries is unclear and should be further explored. These “caries associated” species may be potential biomarkers of early childhood caries in salivary flora.

On the other hand, it is important to point out that *F. periodonticum* and *L.* sp. *oral clone FP036* were detected more frequently in the caries-free group than the caries-affected group in this study. Similarly, Belstrom et al. [35] reported that *F. periodontium* and *Leptotrichia* sp. *clones C3MKM102* and *GT018_ot417/462* were less frequently detected in the caries group as compared to the control group. It is likely that these species might have a protective role against caries.

The limitation of the present study is that due to the constraints of the fieldwork settings and the potential risks of radiation exposure, no radiographs were taken for the detection of dental caries. Some small cavities (particularly those in proximal tooth surfaces) might have been overlooked in the visual and tactile inspection. Furthermore, similar to the previous studies examining the association between bacterial profiles and dental caries [11,24], the sample size in the present study was not enough to reach saturation of the rarefaction curves, which might influence the microbial diversity to a certain extent. Therefore, additional samples might be needed for determining the complete extent of the microbial diversity in saliva.

## 4. Materials and Methods

### 4.1. Sample Size Calculation

The primary outcome of this study was the Shannon index which is the most commonly used microbial diversity statistic [36]. The sample size calculation was performed with reference to a similar study that investigated plaque bacterial microbiome diversity in young children, which detected a mean (SD) of Shannon index as 3.58 (0.02) and 3.55 (0.03) for caries-free and caries-affected subjects, respectively [37]. In order to detect such a difference at a significance level of 5% and a statistical power of 0.90, 17 children in each group were required, and the total sample size needed was 34 children.

### 4.2. Subject Recruitment and Oral Examination

Ethical approval for this study was obtained from the Institutional Review Board of the University of Hong Kong/Hospital Authority Hong Kong West Cluster (#UW 11-483). The subjects were recruited from a kindergarten in Hong Kong. All Chinese children aged 3–4 years old were approached. Parents of 69 children gave written consent. Among them, 17 children were excluded from this study because they had a serious health condition, were under regular medication, refused to cooperate in dental examination or saliva collection, or were absent from school on the examination day. The remaining 52 subjects included 28 caries-free and 24 caries affected children. Among them, 20 caries-free and 20 caries-affected children were selected for this study, with a simultaneous consideration of gender balance (i.e., 10 males and 10 females in each of the caries-free and caries-affected groups).

The tooth status (dental caries) of each child was assessed by using a disposable mouth mirror illuminated by an intraoral light-emitting diode (LED) light. The visual inspection was aided by tactile inspection with a community periodontal index (CPI) probe when necessary. No radiographs were taken. The caries examination method and criteria recommended by the World Health Organization were followed [38]. Decayed teeth were detected at the cavitation level. A trained and calibrated dentist performed all the dental examinations. Duplicate examinations were performed on 7 randomly selected subjects to assess intra-examiner reliability.

### 4.3. Saliva Collection

Prior to saliva collection, the children were asked to refrain from any food, drinks, and tooth brushing for 2 h. Each child was instructed to spit saliva into a sterile container until 2 mL of unstimulated whole saliva sample were collected. Sampling was performed by two dental hygienists. The collected samples were quickly frozen on dry ice, transported to the laboratory within 2 h, and then stored at −80 °C until use [39].

### 4.4. DNA Extraction

Total bacterial DNA was extracted and purified using the QIAmp DNA mini Kit (Qiagen, Hilden, Germany), according to the manufacturer’s instructions. Firstly, 180 μL of lysis buffer (20 mg/mL lysozyme, 50 mM Tris-HCl, pH 8.0, 2 mM EDTA, and 1.2% Triton) was added to suspend the bacterial pellet and was incubated for 1 h at 37 °C. Then, 20 mL of proteinase K and 200 μL of buffer AL were added to the treated saliva sample and were incubated for 90 min at 56 °C and then for a further 15 min at 95 °C. After addition of 200 μL ethanol (96%–100%), the mixture was transferred to the QIAamp Mini spin column and centrifuged for 1 min at 8000 rpm. After that, the DNA pellets were washed with 500 μL Buffer aka Ethanol Wash 1(AW 1) and 500 μL Buffer aka Ethanol Wash 2(AW 2) sequentially. Finally, the DNA was eluted in 200 ml Buffer aka Elution (AE). The quality of the DNA was evaluated by the absorbance measurements at A260/280 using a NanoDrop ND-1000 (Thermo Fisher Scientific Inc., Waltham, MA, USA). The DNA samples were stored at −20 °C before analysis.

### 4.5. PCR Amplification of 16S rRNA Genes and Miseq Sequencing

The V3-V4 hypervariable region of 16S rRNA genes were amplified using universal primers with overhang adapters (338 Fin TTCCCTACACGACGCTCTTCCGATCT-ACTCCTACGGRAGGCAGCAG; 806 Rin GAGTTCCTTGGCACCCGAGAATTCCA-GGACTACHVGGGTWTCTAAT) by the Phusion High Fidelity PCR Master Mix with HF Buffer (New England Biolabs, Hitchin Herts, UK). The PCR reactions were performed as follows: initial denaturation at 94 °C × 2 min; 25 cycles of denaturation at 94 °C × 30 s, annealing at 56 °C × 30 s, elongation at 72 °C × 45 s; and final extension at 72 °C × 2 min and held at 10 °C × 10 min. After purification using the AXYGEN AxyPrep DNA Gel Extraction Kit (Axygen Scientific, Union City, CA, USA), the libraries were then normalized according to Qubit3.0. The barcoded 16S rRNA gene was sequenced on the Illumina MiSeq sequencing platform (Illumnia, Inc., San Diego, CA, USA) at TinyGene Bio-Tech (Shanghai), Co., Ltd. (Shanghai, China), using a 2 × 300 cycle V3 kit, following standard Illumina sequencing protocols.

### 4.6. 16S rRNA Data Analysis

The bacterial 16S rRNA gene sequences were analysed using the MOTHUR software package (V.1.33.3). The paired reads were assembled using make. contigs. Low quality reads were removed by Screen.seqs using filtering parameters as follows; maxambig = 0, minlength = 200, maxlength = 580, and maxhomop = 8. The remaining sequences were simplified to generate a unique set of sequences, and then aligned with the SILVA database (V.119). The chimeras were checked using the UCHIME algorithm and then the chimeric sequences were removed using the chimera.uchime command with default parameters. Then the distance matrix between the aligned sequences was generated by the dist.seqs command. Finally, these sequences were clustered to OTUs at 97% similarity level. The majority consensus taxonomy for each OTU was obtained by the classify.otu command with default parameters.

### 4.7. Statistical Analysis

Rarefaction curves, alpha diversity and richness estimates, and UniFrac distance were calculated by MOTHUR. The core microbiome (OTUs present in all samples) were determined. Student’s *t*-test and Mann-Whitney U test, as appropriate, were used to compare the means of indices of diversity and richness of salivary microbiome, UniFrace distance, and relative abundance using Statistical Package for Social Sciences (version 23.0, SPSS, Chicago, IL, USA). The level of statistical significance was set at 0.05.

## 5. Conclusions

In conclusion, the present study suggests that the caries-free and caries-affected children displayed an overall similar level of highly phylogenetic diversity. There is neither a species-level “core” of salivary microbiome nor a “caries-specific” taxa among preschool children. The current study indicates that the salivary “caries-associated” species (such as *R. dentocariosa*, *A. graevenitzii*, and *F. periodonticum*) may be potential biomarkers for screening and assessing the risk of caries in children.

## Figures and Tables

**Table 1:** Sequences of individual saliva samples of the caries-affected (CA) and caries-free (CF) children.

Subject Code	dmft	Gender	Age	Reads after Trimming	OTUs
CA 1	2	Male	3	25,576	1974
CA 2	2	Male	3	16,157	2452
CA 3	2	Female	4	18,200	2306
CA 4	2	Female	3	19,108	2304
CA 5	10	Male	4	18,413	1919
CA 6	16	Female	3	17,718	2123
CA 7	2	Male	3	16,232	2591
CA 8	2	Male	3	18,760	2445
CA 9	6	Female	4	19,230	2201
CA 10	7	Male	3	16,157	2770
CA 11	2	Female	3	17,075	2254
CA 12	2	Male	3	17,786	2403
CA 13	2	Male	4	16,123	2306
CA 14	2	Female	4	15,562	2310
CA 15	2	Male	3	17,903	2535
CA 16	2	Female	3	17,142	2225
CA 17	2	Male	3	19,079	2190
CA 18	2	Female	4	17,764	2398
CA 19	2	Female	3	18,463	2476
CA 20	10	Female	3	16,591	2134
CF 1	0	Male	3	27,683	1979
CF 2	0	Male	3	29,705	2306
CF 3	0	Male	4	18,148	1559
CF 4	0	Female	3	19,484	2025
CF 5	0	Female	3	19,253	2370
CF 6	0	Female	3	17,744	2212
CF 7	0	Male	4	20,320	2639
CF 8	0	Male	3	19,140	2376
CF 9	0	Female	4	17,343	2127
CF 10	0	Female	3	18,482	2133
CF 11	0	Male	3	19,452	1796
CF 12	0	Male	4	18,148	2130
CF 13	0	Male	4	18,538	2259
CF 14	0	Male	4	18,751	2416
CF 15	0	Female	3	18,602	2122
CF 16	0	Female	3	17,385	2406
CF 17	0	Female	3	18,353	2334
CF 18	0	Female	3	18,456	2702
CF 19	0	Male	3	16,986	2610
CF 20	0	Female	4	16,582	2684

dmft: decayed, missing and filled teeth; OTUs: Operational Taxonomic Units (defined with 3% dissimilarity).

## Abbreviations

ACE	Abundance-based coverage estimator
AW	Aka Ethanol Wash
AE	Aka Ethanol
CA	Caries-affected
CF	Caries-free
dmft	Decayed, missing and filled teeth
HOMIM	Human Oral Microbe Identification Microarray
LED	Light-emitting diode
OTU	Operational taxonomic units
PCR-DGGE	Polymerase chain reaction denaturing gradient gel electrophoresis
